# Relationship of Hematological Profiles with the Serum Complement System in Patients with Systemic Lupus Erythematosus

**DOI:** 10.3390/biomedicines12050967

**Published:** 2024-04-27

**Authors:** Yolanda Fernández-Cladera, María García-González, Marta Hernández-Díaz, Fuensanta Gómez-Bernal, Juan C. Quevedo-Abeledo, Agustín F. González-Rivero, Antonia de Vera-González, Cristina Gómez-Moreno, Miguel Á. González-Gay, Iván Ferraz-Amaro

**Affiliations:** 1 Division of Central Laboratory, Hospital Universitario de Canarias, 38320 Tenerife, Spain; yolanda.fernandezcladera@gmail.com (Y.F.-C.); fuensanta95@gmail.com (F.G.-B.); afgonriv@gmail.com (A.F.G.-R.); adeverag@gmail.com (A.d.V.-G.); 2 Division of Rheumatology, Hospital Universitario de Canarias, 38320 Tenerife, Spain; margagon23@hotmail.com (M.G.-G.); martahediaz@gmail.com (M.H.-D.); 3 Division of Rheumatology, Hospital Doctor Negrín, 35010 Las Palmas de Gran Canaria, Spain; quevedojcarlos@yahoo.es; 4 School of Nursing, Fundación Jiménez Díaz, Autonomous University of Madrid, 28040 Madrid, Spain; cgomezm@fjd.es; 5 Division of Rheumatology, Instituto de Investigación Sanitaria Fundación Jiménez Díaz, 28040 Madrid, Spain; 6 Department of Internal Medicine, University of Cantabria, 39005 Santander, Spain; 7 Department of Internal Medicine, University of La Laguna (ULL), 38200 Tenerife, Spain

**Keywords:** complement system, systemic lupus erythematosus, blood cells count, hemoglobin, leucocytes, neutrophils, monocytes, lymphocytes, platelets

## Abstract

Systemic lupus erythematosus (SLE) is a chronic autoimmune disorder identified by hematological abnormalities including anemia, leukopenia, and thrombocytopenia. Complement system disturbance is implicated in the pathogenesis of SLE. In this work, we aim to study how a full assessment of the complement system, which includes the evaluation of its three pathways, relates to blood cell counts in a population of patients with SLE. New-generation functional assays of the classical, alternative, and lectin pathways of the complement system were conducted in 284 patients with SLE. Additionally, serum levels of inactive molecules (C1q, C2, C3, C4, factor D) and activated molecules (C3a), as well as regulators (C1-inhibitor and factor H), were evaluated. Complete blood cell counts were analyzed. Multivariable linear regression analysis was performed to study the relationship of hematological profiles with this full characterization of the complement system. After multivariable adjustments that included age, sex, SLICC-DI (damage), and SLEDAI (activity) scores, as well as the use of aspirin, prednisone, methotrexate, azathioprine, and mycophenolate mofetil, several relationships were observed between the C pathways and the individual products and blood cells profile. Lower values of C1q and C2 were associated with lower hemoglobin levels. Lower leukocyte counts showed significantly lower values of C4, C1 inhibitor, C3, factor D, and alternative pathway functional levels. Neutrophil counts showed significant negative relationships only with the alternative pathway and C1-inh. In the case of lymphocytes, associations were found, especially with functional tests of the classical and alternative pathways, as well as with C2, C4, C3, and C3a. On the contrary, for platelets, significance was only observed, after multivariable adjustment, with lower C2 concentrations. In conclusion, the serum complement system and hematological profile in SLE are independently linked, after adjustment for disease activity and damage. These relationships are basically negative and are predominantly found in lymphocytes.

## 1. Introduction

Systemic lupus erythematosus (SLE) is a chronic autoimmune condition marked by dysregulated immunity, capable of impacting various organs such as the skin, joints, kidneys, and the cardiovascular and central nervous systems. Clinical manifestations range from mild to severe, encompassing symptoms such as fatigue, arthralgia, mucocutaneous manifestations, constitutional symptoms, and organ-specific complications. Hematologic abnormalities are common in SLE, manifesting at both diagnosis and during the course of the disease. Key hematologic manifestations of SLE encompass anemia, leukopenia, thrombocytopenia, lymphadenopathy, or splenomegaly [1]. These abnormalities affect the three-blood component series and may be a manifestation of SLE, be associated with another concurrent condition, and/or emerge as a consequence of SLE treatment [2]. In this context, anemia affects over half of individuals with SLE. Various factors can contribute to anemia, including chronic inflammation, iron deficiency, medications, autoimmune hemolysis, vitamin B12 deficiency, and thrombotic microangiopathies [3]. Leukopenia is also common in SLE and often correlates with disease activity [4]. Neutropenia can be induced by immunosuppressive medications or hypersplenism, while lymphocytopenia may result from autoantibodies [5]. Lastly, mild thrombocytopenia is often seen in SLE [6]. Immune thrombocytopenia due to autoimmune platelet destruction may present as a chronic complication or acutely during a disease flare. Other causes of thrombocytopenia include medications, splenomegaly, a thrombotic microangiopathy, or antiphospholipid syndrome [7].

The complement (C) system plays a critical role in innate immunity, particularly in antibody-triggered responses. It comprises approximately 60 plasma and membrane proteins organized into three distinct, yet interconnected, activating pathways (classical, alternative, and lectin pathways), along with a shared terminal lytic cascade and a network of regulators and receptors [8]. For decades, it has been known that C is implicated in the pathogenesis of SLE [9]. This is supported by various types of evidence. For instance, homozygous deficiency of any of the proteins within the classical pathway is causally associated with susceptibility to the development of SLE, particularly deficiency of the earliest proteins in the activation pathway [10]. Additionally, C is involved in the effector inflammatory phase of the autoimmune response characteristic of the disease, and C proteins are deposited in inflamed tissues, causing injury [11]. Likewise, the disease processes in SLE lead to the development of autoantibodies against certain C proteins [12]. Moreover, hypocomplementemia is a typical laboratory finding in patients with SLE, often indicating activation of the C system by immune complexes [13].

The relationship between blood abnormalities and the C system in SLE have been mainly studied in the context of hemolytic anemia and antiphospholipid syndrome. In this regard, in the hemolytic anemia of SLE, IgG subtype autoantibodies typically attack red cells, allowing the C membrane attack complex to be deposited on the red blood cell surface [14]. Moreover, the activation of C is a major driver of the vascular and obstetric complications of antiphospholipid syndrome [15]. However, there is a gap in the existing literature regarding the comprehensive analysis of the serum C system, as well as a complete hematological profile in patients with SLE. To close this gap, we performed functional measurements of all three C pathways, as well as specific particles associated with these cascades, including active and inactive particles and regulators. Our objective was to examine the correlation between this comprehensive characterization of the serum C system and blood cell counts in a well-characterized cohort of SLE patients that included assessments of disease activity and damage.

## 2. Materials and Methods

### 2.1. Study Participants

This was a cross-sectional study that involved 284 patients diagnosed with SLE. All participants were aged 18 years or older, were clinically diagnosed with SLE, and met at least four of the American College of Rheumatology (ACR) classification criteria for SLE [16]. They were diagnosed by rheumatologists and were regularly monitored in rheumatology outpatient clinics. To qualify for the study, patients were required to have a disease duration exceeding 1 year. Exclusion criteria included a history of cancer, chronic liver and/or renal failure, signs of acute and/or chronic active infection, and/or any other chronic autoimmune disease aside from conditions like antiphospholipid and/or Sjögren’s syndrome associated with SLE. None of the patients had aplasia, myeloproliferative disorders, or any other primary hematological diseases. The research adhered to the principles outlined in the Declaration of Helsinki. The study protocol was approved by the Institutional Ethics Committees of the Hospital Universitario de Canarias and the Hospital Universitario Doctor Negrín (both in Spain), and all subjects provided informed written consent (Approval Number 2020-123).

### 2.2. Data Collection

Patients enrolled in the study completed a questionnaire detailing their medication usage and underwent a thorough physical examination. Medical records were meticulously reviewed to confirm specific diagnoses and prescribed medications. The assessment of SLE disease activity and damage was guided using the Systemic Lupus Erythematosus Disease Activity Index-2000 (SLEDAI-2K) [17] and the Systemic Lupus International Collaborating Clinics/American College of Rheumatology (SLICC/ACR) Damage Index (SDI) [18], respectively. The SLEDAI-2k index was divided into none (0 points), mild (1–5 points), moderate (6–10 points), high (11–19), and very high activity (>20) as previously described [19]. 

### 2.3. Laboratory Assessments

Blood cell counts were measured using the Sysmex-XN automated blood cell analyzer (Sysmex, Kobe, Japan). This instrument uses the standard deviation, rather than the coefficient of variation, of the mean corpuscular volume distribution curve to calculate the red cell distribution width. The Wieslab^®^ brand (SVAR Life Sciences, Malmö, Sweden) functional C assays were used to assess classical, alternative, and lectin pathway activity. These tests combine principles of the hemolytic assay for C function with the use of labeled antibodies specific for the neoantigen produced as the result of C activation. The amount of neoantigen generated is proportional to the functional activity of the C pathways. Microtiter strip wells are coated with activators specific to the classical, alternative, or lectin pathways of the C system. The patient’s serum is diluted in a diluent containing a specific blocker to ensure activation of only the pathway under study. Subsequently, during incubation of the diluted patient serum in the wells, the specific coating activates C. After washing the wells, detection of C5b-9 is achieved using an alkaline phosphatase-labeled specific antibody targeting the neoantigen expressed during membrane attack complex (MAC) formation. After an additional washing step, the detection of specific antibodies is achieved by incubation with alkaline phosphatase substrate solution. The amount of C activation correlates with the intensity of the color and is measured in terms of absorbance (optical density). The level of formed MAC (neo-epitope) reflects the activity of the C cascade. For these functional tests, lower values of classical, alternative, and lectin cascade indicate higher activation of the respective pathway. The results are expressed semi-quantitatively using the optical density ratio between a positive control and the sample. Wieslab^®^ has validated these functional assays by studying their correlation and concordance with the classical CH50 and AH50 hemolytic tests (https://www.svarlifescience.com/ accessed on 10 February 2024). C2, C3, C3a, C4, and C1q were analyzed by turbidimetry (Roche Diagnostics, Barcelona, Spain), the C1-inhibitor was analyzed through nephelometry (Siemens, Marburg, Germany), while factor D and factor H were assessed by enzyme-linked immunosorbent assay (ELISA Duoset, R&D, Minneapolis, USA). Both intra- and inter-coefficients of variability were <10% for these assays.

### 2.4. Statistical Analysis

Demographic and clinical characteristics were described as mean ± standard deviation (SD) or percentages for categorical variables. Non-normally distributed continuous variables were presented as the median and interquartile range (IQR). The normality of the distribution of the quantitative variables was tested using histograms, P-P plots, and the Shapiro–Wilk test. The relationship between the SLE blood cell counts and the C pathways and products was assessed through multivariable linear regression analysis. For this, hematological cell counts were categorized in terciles. All the analyses used a 5% two-sided significance level and were performed using Stata software, version 17/SE (StataCorp, College Station, TX, USA). *p*-values <0.05 were considered statistically significant.

## 3. Results

### 3.1. Demographic and Disease-Related Data of Patients with Systemic Lupus Erythematosus

The demographic and disease-related characteristics of SLE patients are presented in Table 1. The majority were female (92%), with a mean age of 50 ± 12 years. The average age at diagnosis was 34 ± 13 years, and the disease duration was 16 ± 10 years. Upon enrollment, 67% of patients tested positive for anti-DNA antibodies, and 69% for ENA, with anti-SSA being the most commonly detected antibody (35%). A total of 16% of patients met the criteria for associated antiphospholipid syndrome, and 32% had at least one positive antiphospholipid antibody. Most SLE patients were categorized as having no activity (40%) or mild-moderate activity (55%), as indicated by the SLEDAI-2K score. The SDI was 1 (IQR 0-2), with 68% of patients having an SDI score of 1 or higher.

At the time of assessment, half of the patients (50%) were receiving glucocorticoids, with a median equivalent daily dose of prednisone of 5 mg/day (IQR 5–7.5 mg). Additionally, 69% of patients were prescribed hydroxychloroquine at the time of the study. Less commonly used medications included methotrexate (11%) and azathioprine (15%). Table 1 shows additional information on the data related to SLE.

Functional C assays of the classical, alternative, and lectin pathways were 91 ± 38%, 41 (IQR 12–79)% and 10 (IQR 1–41)%, respectively. the single C components, C1q, C2, C3, C3a, C1-inhibitor (C1-inh), and the factor D and H serum values are shown in Supplementary Table S1. Additionally, the blood cell counts of the three series—red blood cells, white blood cells, and platelets—are provided in Supplementary Table S1.

### 3.2. Blood Cells Counts Univariable Relationship with Complement System Pathways and Elements

**Figure 1:**
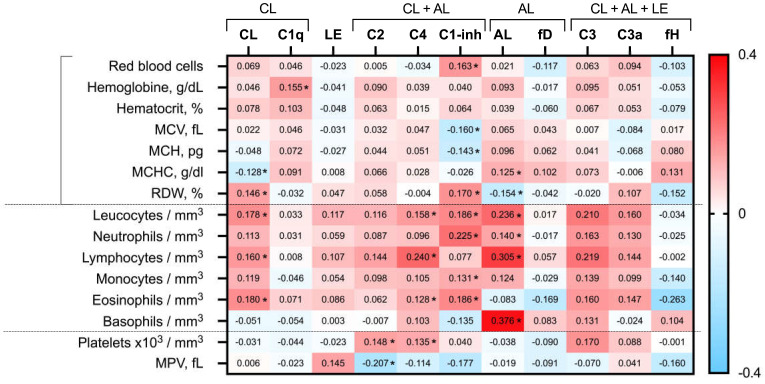
Heatmap of blood cell count relationship with the complement functional routes assays and serum molecules. Values in the cells represent Spearman’s rho coefficient (* denotes *p* value < 0.05). Positive and negative correlations are shown in red and blue, respectively. fD: factor D; fH: factor H. CL, LE, and AL refer to the functional tests of these cascades: classical (CL), lectin (LE), and alternative (AL).

Figure 1 depicts the univariable analysis examining the relationship between the C pathways and their respective proteins with the complete hematological cell profile. Concerning red blood cells, the notable associations were limited. Specifically, the hemoglobin levels showed a significant and positive correlation solely with C1q values, while the hematocrit did not demonstrate significant associations with any C values. Additionally, the mean corpuscular volume (MCV) and mean corpuscular hemoglobin (MCH) values displayed a significant and negative correlation with the C1-inhibitor, whereas the mean corpuscular hemoglobin concentration (MCHC) exhibited a negative correlation with the classical pathway, but a positive correlation with the alternative cascade. Conversely, red cell distribution width (RDW) demonstrated a positive correlation with classical pathway levels and C1-inh, but a negative correlation with the alternative route and factor H.

On the contrary, there were numerous and predominantly positive relationships (indicated in red on the heatmap) between the white blood cell series and the C values (Figure 1). Specifically, the leukocyte count exhibited significant and positive correlations with the functional test results of both the classical and alternative pathways, as well as with the proteins C4, C1-inhibitor, C3, and C3a. Similarly, the lymphocytes displayed significant and positive relationships with the classical and alternative pathway tests, as well as with the values of C2, C4, C3, and C3a. Regarding the monocytes, they demonstrated significant positive relationships with the functional test of the alternative pathway, C1-inhibitor, and C3, but exhibited a negative correlation with factor H. Figure 1 illustrates additional relationships with the neutrophils, eosinophils, and basophils. Concerning platelets, their levels exhibited a significant and positive correlation with the values of C2, C3, and C4. Likewise, the mean platelet volume (MPV) values, which typically have an inverse correlation with platelet counts, displayed significant and negative relationships with C2 and C1-inhibitor (Figure 1). 

A comprehensive breakdown of the heatmap correlations is provided in Supplementary Table S1.

### 3.3. Multivariable Analysis of the Relationship of Lymphocytes, Neutrophils, Hemoglobin, and Platelets with the Complement System

Considering that blood cell values and C levels can be influenced by various factors, such as disease activity and medication intake, we conducted a multivariable analysis adjusted for age, sex, SLICC (damage), and SLEDAI (activity) scores, as well as for the use of aspirin, prednisone, methotrexate, azathioprine, and mycophenolate mofetil. For the SLEDAI score, items related to hypocomplementemia, anti-DNA positivity, leukopenia, and thrombocytopenia were subtracted from its calculation.

**Figure 2:**
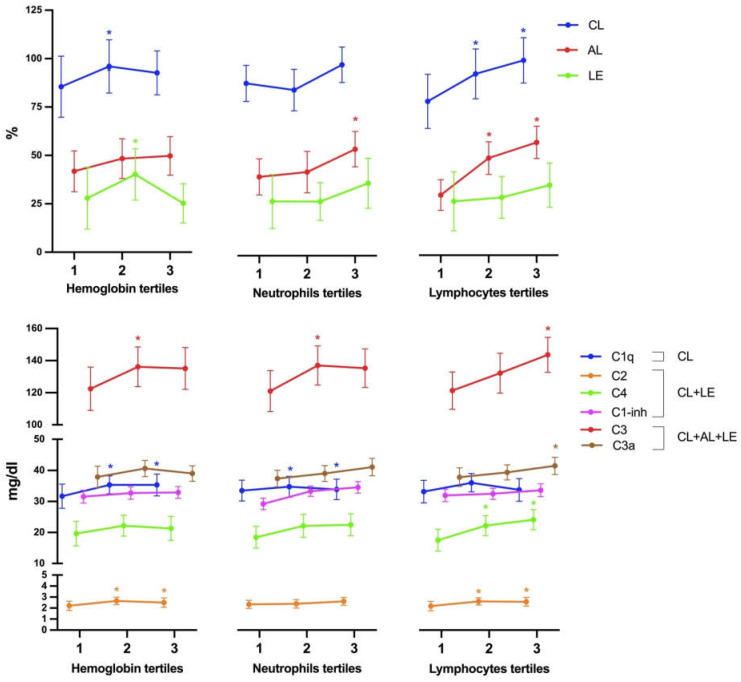
Classical (CL), lectin (LE), and alternative (AL) functional assays values (**top**) and complement products (**bottom**) are showed, divided by terciles of hemoglobin, neutrophils, and lymphocytes (**top**). Significant differences between the 2nd and 3rd terciles compared to the 1st tercile (which is considered the reference category) are presented as *. Factor D and H values are not shown in this figure. Data shown are adjusted for age, sex, SLICC-DI (damage), and SLEDAI (activity) scores, as well as for the use of aspirin, prednisone, methotrexate, azathioprine, and mycophenolate mofetil.

Regarding hemoglobin levels, patients in the third tercile demonstrated higher values of C1q and C2 compared to subjects in the first tercile (reference category), after adjusting for the aforementioned covariates (Figure 2 and Supplementary Table S3). In terms of leukocyte count, numerous significant comparisons were identified. Specifically, SLE patients in the third tercile of leukocytes exhibited significantly elevated values of C4, C1-inhibitor, C3, factor D, and functional levels of the alternative pathway, after adjusting for confounding factors. Similar associations were observed for lymphocytes, particularly with the functional tests of the classical and alternative pathways, as well as with C2, C4, C3, and C3a. Neutrophil counts displayed significant relationships solely with the alternative pathway and C1-inh. Conversely, for platelets, significance was only observed after multivariable adjustment with C2 (Figure 2 and Supplementary Table S3). Although cytopenias were not frequent in our series of patients, the differences between patients with or without these cytopenias in their C values were similar to the data shown by the terciles (Supplementary Table S4).

## 4. Discussion

Our study represents the most comprehensive investigation to date exploring the relationship between the hematologic profile of patients with SLE and a thorough characterization of the three C system pathways in serum. Our findings reveal a strong association between these factors, which cannot be solely explained by disease activity, damage, or the treatments administered for the disease. Notably, lymphocytes emerge as the cell line exhibiting the strongest correlation within this relationship.

Anemia affects over a half of individuals with SLE [3]. Multiple factors may contribute to its development, including chronic inflammation, iron deficiency, medications, autoimmune hemolysis, and vitamin B12 deficiency. Traditionally, the association between the C system and anemia has been observed primarily when hemolytic anemia coexists in SLE [14]. None of the patients included in our study exhibited active hemolytic anemia at the time of enrollment. Our findings suggest that the relationship between the C system and red blood cells appeared to be weaker compared to its association with white blood cells. After adjusting for multiple variables, we identified a relationship between hemoglobin levels and C2 and C1q, which are upstream particles of the classical cascade. This association was positive, indicating that lower or depleted levels of C2 and C1q were linked to lower hemoglobin levels. Remarkably, hemoglobin did not demonstrate a significant relationship to any of the functional C assays of the three pathways. Furthermore, C3a did not display significant associations with any of the red cell values. Based on these findings, we suggest that red cell count is not associated with a specific activation state of the serum C system cascades.

A reduced white blood cell count is a common occurrence in SLE and often correlates with disease activity [20]. Neutrophil dysfunction is believed to be induced by immune abnormalities such as immune complexes, inhibition of complement-derived chemotactic factors, and medications. Previous data suggest that the mild neutropenia found in about half of SLE patients is caused by the binding of antibodies to neutrophils, leading to subsequent C fixation and peripheral destruction of the cells [21]. In our study, we observed a significant univariable relationship between the neutrophil values and C3, C3a, the functional test for the alternative pathway, and C1-inhibitor. After adjustment, lower levels of C1-inhibitor and activation of the alternative cascade were associated with decreased neutrophil counts. In terms of the lymphocytes, the relationships were stronger. In this regard, their levels were negatively associated with activation of the alternative and classical pathways after adjusting for covariates. Additionally, the presence of lymphocytopenia was associated with lower levels of C2, C4, and C3 and C3a. It is noteworthy that this adjustment included disease activity and damage. In vitro studies have indicated that lymphocytopenia might result from autoantibodies targeting the lymphocytes. Additionally, these autoantibodies are correlated with hypocomplementemia, suggesting a complement-mediated cytotoxicity [22]. According to these findings, serum C values are strongly associated with white blood cell counts in patients with SLE. These associations generally encompass the classical and alternative pathways, rather than the lectin cascade, and involve both upstream and downstream components of these pathways, including C3a.

In our study, platelet counts demonstrated correlations with C2, C3, and C4 in the univariable analysis. However, after adjusting for disease damage and activity, as well as for the medications used in the treatment of the disease, the significant relationships with C3 and C4 were no longer present. Remarkably, none of the functional tests of the three C pathways, nor other individual products, exhibited significant associations with platelet counts. In the general population, it is recognized that localized C activation enhances the procoagulant responses of platelets by inducing the generation of procoagulant microparticles through the insertion of sublytic amounts of C5b-9 into the platelet membrane [23]. The primary mechanism implicated in immune-mediated thrombocytopenia in SLE is proposed to involve immunoglobulin binding to platelets, followed by phagocytosis in the spleen [24]. In our study, the relationship between the C system and platelet values was found to be weak. Consequently, we conclude that the C system assessed in serum is not associated with platelet values in patients with SLE.

In a previous study conducted by our group, we performed a comprehensive characterization of the components of the three C pathways in patients with SLE, examining their relationship with disease activity, severity, and damage [13]. In that study, we found that not only the classical pathway, but also the alternative and lectin pathways, were associated with SLE features. Consequently, patterns of C expression were correlated with disease profiles. While accumulated damage was linked to higher functional tests of C pathways, anti-DNA, anti-ribosomes, and anti-nucleosomes antibodies showed a stronger association with C activation, primarily through the lectin and classical cascades [13]. However, in that study, we did not investigate how C expression was linked to the hematological abnormalities observed in the disease. This work delves deeper into the relationship between the C system and the hematological disturbances exhibited by the disease.

Our study possesses the strength of having measured a complete hematological profile that includes its three-component series and having conducted an in-depth characterization of the C system, which includes the three cascades. Some relationships may appear to have a small effect. However, the beta coefficients depend on the unit of measurement of the independent and dependent variables. Additionally, a seemingly small effect may prove to large after all, depending on the true pathophysiological mechanism underlying that relationship. We acknowledge several limitations in our study. Firstly, being cross-sectional in nature, causality cannot be inferred. Prospective studies will be necessary to analyze the relationship between the C system and the hematological profile over the course of the disease. Additionally, iron levels were not assessed in our cohort. If the C system were related to iron values, this could potentially impact our results. However, it has not been previously documented that there is a relationship between iron deposits and the C system. Therefore, we believe that this possibility is remote. Additionally, because our primary aim was to investigate the relationship between blood cells and the C system measured in serum, it cannot be ruled out that other relationships may exist at the cellular level. Lastly, we did not recruit controls. Therefore, we cannot draw conclusions about how this relationship manifests in healthy subjects who are not affected by an immune-mediated disease.

## 5. Conclusions

In conclusion, serum C system values and blood cell count are correlated in patients with SLE. This relationship is independent of disease damage and activity, and it is predominantly observed for the lymphocytes.

## Figures and Tables

**Table 1:** Demographic and disease-related data of patients.

	SLE
	(*n* = 284)
Age, years	50 ± 12
Women, *n* (%)	261 (92)
Diabetes, *n* (%)	16 (6)
Smoking, *n* (%)	69 (24)
Obesity, *n* (%)	85 (30)
Hypertension, *n* (%)	111 (39)
SLE-related data	
Age at diagnosis, years	34 ± 13
Disease duration, years	16 ± 10
Auto-antibody profile	
Anti-DNA positive, *n* (%)	151 (67)
ENA positive, *n* (%)	164 (69)
Anti-Sm	24 (10)
Anti-ribosome	13 (9)
Anti-nucleosome	32 (22)
Anti-RNP	64 (28)
Anti-histone	22 (15)
Anti-SSA/Ro	55 (35)
Anti-SSB/La	36 (21)
ACA IgM	22 (11)
ACA IgG	39 (20)
Antiphospholipid syndrome, *n* (%)	43 (16)
Any antiphospholipid antibody, *n* (%)	61 (32)
Anti beta2 glycoprotein IgM	19 (10)
Anti beta2 glycoprotein IgG	28 (15)
Disease scores	
Median SLEDAI-2K	2 (0–4)
SLEDAI-2K categories, *n* (%)	
No activity	109 (40)
Mild	107 (40)
Moderate	41 (15)
High or very high	14 (5)
Median SDI	1 (0-2)
SDI ≥ 1, *n* (%)	191 (68)
Immunosuppressants at the time of the visit	
Glucocorticoids, *n* (%)	140 (50)
Methotrexate, *n* (%)	31 (11)
Prednisone equivalent daily dose, mg	5 (5–7.5)
Antimalarials drugs, *n* (%)	194 (69)
Azathioprine, *n* (%)	43 (15)
Mycophenolate mofetil, *n* (%)	31 (11)
Antimalarials + methotrexate, *n* (%)	21 (8)
Antimalarials + azathioprine, *n* (%)	27 (10)
Antimalarials + Mycophenolate mofetil, *n* (%)	21 (8)
Belimumab, *n* (%)	8 (3)
Rituximab, *n* (%)	8 (3)

Data represent mean ± SD or median (interquartile range) when data were not normally distributed. SLE: systemic lupus erythematosus. SLEDAI-2K: SLE Disease Activity Index. SLEDAI categories were defined as: 0, no activity; 1–5, mild; 6–10, moderate; >10 active. ACA: anticardiolipin antibodies; ENA: extractable nuclear antibodies; SDI: SLICC/ACR Damage Index; SLE: systemic lupus erythematosus.

## Data Availability

The datasets used and/or analyzed in the present study are available from the corresponding author upon request. The data are not publicly available due to privacy and ethical restrictions.

## Supplementary Materials

The following supporting information can be downloaded at: https://www.mdpi.com/article/10.3390/biomedicines12050967/s1, Table S1: Complement system and hematological cell count values. Table S2: Correlations between hematological cells and complement functional pathways and products. Table S3: Multivariable analysis of the relationship of lymphocytes, neutrophils, hemoglobin, and platelets with the complement system. Table S4: Multivariable relationship of cytopenias to the presence of anemia, leukopenia, neutropenia, lymphopenia, and thrombocytopenia.
