# Supplementary material for: Relationship of Hematological Profiles with the Serum Complement System in Patients with Systemic Lupus Erythematosus
Source: Biomedicines. 2024 Apr 27;12(5):967. doi: 10.3390/biomedicines12050967 (PMC11117834; doi:10.3390/biomedicines12050967)
Supplement: Supplementary file 1 [file biomedicines-12-00967-s001.zip › biomedicines-2951956-supplementary.pdf]

Supplementary Table S1. Complement system and hematological cell count values.

	SLE patients (n=284)
Hematological cells count	
Red blood cells, x10e6/mm3	4.44 ± 0.43
Hemoglobin, g/dL	13.2 ± 1.3
Hematocrit, %	40.1 ± 3.7
Mean corpuscular volume, fL	90.6 ± 6.1
Mean corpuscular hemoglobin, pg	29.9 ± 2.5
Mean corpuscular hemoglobin concentration, g/dl	33.0 ± 1.2
Red Cell Distribution Width, %	13.8 ± 2.1
Leucocytes / mm3	6042 ± 2276
Neutrophils / mm3	3524 ± 1733
Lymphocytes / mm3	1787 ± 957
Monocytes / mm3	548 ± 208
Eosinophils / mm3	70 (30-140)
Basophils / mm3	40 (20-100)
Platelets x10e3 / mm3	237 ± 79
Mean platelet volume, fL	9.6 ± 1.6
Complement system functional assays and proteins	
Functional assays, %	
Classical pathway	91 ± 38
Alternative pathway	41 (12-79)
Lectin pathway	10 (1-41)
Individual complement components	
C1q, mg/dl	34 ± 11
C2, mg/dl	2.5 ± 1.2
C4, mg/dl	21 ± 12
Factor D, ng/ml	2593 ± 1835
C3, mg/dl	130 ± 40
C3a, mg/dl	39 ± 10
C1 inhibitor, mg/dl	32 ± 9
Factor H, ng/ml x10e-3	388 (281-564)

Supplementary Table S2. Correlations between hematological cells and complement functional pathways and products

		Classical		Lectin	Classical and lectin			Alternative		Classical, alternative and lectin		
		%	C1q	%	C2	C4	C1-inh	%	Factor D	C3	C3a	Factor H
Red cells, x10e6/mm3	Rho	0.0691	0.0461	-0.0229	0.0048	-0.0342	**0.1630**	0.0206	-0.1169	0.0627	0.0939	-0.1026
	p	0.2672	0.4573	0.7126	0.9382	0.5928	**0.0137**	0.7409	0.0902	0.3244	0.1289	0.1373
Hemoglobin, g/dL	Rho	0.0463	**0.1554**	-0.0411	0.0903	0.0389	0.0404	0.0931	-0.0167	0.0953	0.0505	-0.0529
	p	0.4571	**0.0118**	0.5091	0.1417	0.5426	0.5443	0.1342	0.8092	0.1335	0.4145	0.4444
Hematocrit, %	Rho	0.0782	0.1034	-0.0484	0.0632	0.0153	0.0641	0.0388	-0.0595	0.0674	0.0531	-0.0792
	p	0.2088	0.0948	0.4373	0.3046	0.8114	0.3354	0.5332	0.3897	0.2893	0.3907	0.2522
MCV, fL	Rho	0.0216	0.0463	-0.0306	0.0321	0.0470	**-0.1601**	0.0652	0.0428	0.0072	-0.0840	0.0165
	p	0.7283	0.4559	0.6231	0.6024	0.4624	**0.0156**	0.2949	0.5366	0.9096	0.1746	0.8113
MCH, pg	Rho	-0.0481	0.0720	-0.0271	0.0439	0.0506	**-0.1433**	0.0958	0.0622	0.0406	-0.0677	0.0802
	p	0.4398	0.2453	0.6634	0.4755	0.4284	**0.0305**	0.1235	0.3685	0.5235	0.2739	0.2459
MCHC, g/dl	Rho	**-0.1280**	0.0911	0.0080	0.0658	0.0283	-0.0262	**0.1252**	0.1017	0.0726	-0.0056	0.1310
	p	**0.0391**	0.1415	0.8981	0.2853	0.6578	0.6938	**0.0437**	0.1410	0.2538	0.9280	0.0574
RDW, %	Rho	**0.1461**	-0.0315	0.0468	0.0578	-0.0040	**0.1704**	**-0.1544**	-0.0418	-0.0200	0.1070	**-0.1522**
	p	**0.0185**	0.6117	0.4522	0.3480	0.9498	**0.0099**	**0.0127**	0.5463	0.7534	0.0833	**0.0271**
Leucocytes / mm3	Rho	**0.1783**	0.0327	0.1168	0.1162	**0.1577**	**0.1861**	**0.2360**	0.0165	**0.2104**	**0.1597**	-0.0337
	p	**0.0039**	0.5983	0.0600	0.0584	**0.0131**	**0.0048**	**0.0001**	0.8114	**0.0008**	**0.0095**	0.6269
Neutrophils / mm3	Rho	0.1133	0.0314	0.0591	0.0872	0.0961	**0.2254**	**0.1404**	-0.0166	**0.1634**	**0.1299**	-0.0246
	p	0.0681	0.6130	0.3421	0.1562	0.1322	**0.0006**	**0.0235**	0.8108	**0.0098**	**0.0352**	0.7222
Lymphocytes / mm3	Rho	**0.1601**	0.0079	0.1065	**0.1440**	**0.2403**	0.0769	**0.3054**	0.0572	**0.2193**	**0.1443**	-0.0019
	p	**0.0097**	0.8983	0.0866	**0.0188**	**0.0001**	0.2477	**0.0000**	0.4081	**0.0005**	**0.0192**	0.9777
Monocytes / mm3	Rho	0.1189	-0.0460	0.0539	0.0982	0.1054	**0.1309**	**0.1244**	-0.0287	**0.1385**	0.0986	**-0.1397**
	p	0.0555	0.4588	0.3872	0.1100	0.0983	**0.0484**	**0.0451**	0.6782	**0.0289**	0.1107	**0.0426**
Eosinophils / mm3	Rho	**0.1801**	0.0711	0.0862	0.0621	**0.1277**	**0.1856**	-0.0829	**-0.1689**	**0.1604**	**0.1472**	**-0.2633**
	p	**0.0036**	0.2515	0.1658	0.3128	**0.0449**	**0.0049**	0.1825	**0.0140**	**0.0113**	**0.0169**	**0.0001**
Basophils / mm3	Rho	-0.0506	-0.0541	0.0025	-0.0066	0.1029	**-0.1352**	**0.3763**	0.0830	**0.1310**	-0.0239	0.1039
	p	0.4161	0.3829	0.9680	0.9151	0.1067	**0.0413**	**0.0000**	0.2302	**0.0388**	0.6992	0.1326
Platelets x10e3 / mm3	Rho	-0.0312	-0.0436	-0.0231	**0.1475**	**0.1347**	0.0402	-0.0375	-0.0899	**0.1700**	0.0879	-0.0006
	p	0.6166	0.4820	0.7103	**0.0161**	**0.0344**	0.5460	0.5473	0.1934	**0.0072**	0.1553	0.9930
MPV, fL	Rho	0.0061	-0.0228	0.1451	**-0.2070**	-0.1139	**-0.1771**	-0.0191	-0.0906	-0.0698	0.0410	-0.1595
	p	0.9369	0.7647	0.0597	**0.0057**	0.1416	**0.0313**	0.8049	0.2944	0.3658	0.5915	0.0636

Significant p values are depicted in bold. Correlation Spearman Rho coefficients are shown.

Supplementary Table S3. Multivariable analysis of the relation of lymphocytes, neutrophils, hemoglobin and platelets with complement system

	Beta coefficient (95% confidence interval), p					
	Classical		Lectin	Classical and lectin		
	%	C1q	%	C2	C4	C1-inh
Hemoglobin						
1st tercile	-	-	-	-	-	-
2nd tercile	**12 (0.5-24), 0.040**	**4 (1-7), 0.009**	**17 (4-30), 0.011**	**0.5 (-0.1-0.8), 0.006**	2 (-2-6), 0.29	2 (-1-5), 0.32
3rd tercile	7 (-5-20), 0.23	**5 (1-8), 0.009**	0.05 (-14-14), 0.99	**0.4 (0.03-0.8), 0.033**	2 (-3-6), 0.45	2 (-2-5), 0.33
Leucocytes						
1st tercile	-	-	-	-	-	-
2nd tercile	6 (-6-17), 0.32	1 (-2-4), 0.53	11 (-3-24), 0.12	0.2 (-0.2-0.6), 0.27	**4 (0.2-8), 0.037**	**4 (0.5-7), 0.024**
3rd tercile	11 (-0.6-23), 0.063	-1 (-5-2), 0.41	14 (0.03-27), 0.050	0.1 (-0.2-0.5), 0.44	**4 (0.05-8), 0.047**	**4 (0.9-7), 0.011**
Neutrophils						
1st tercile	-	-	-	-	-	-
2nd tercile	-3 (-15-9), 0.60	1 (-2-4), 0.57	2 (-12-15), 0.82	0.06 (-0.3-0.4), 0.75	3 (-0.8-7), 0.12	**4 (1-7), 0.006**
3rd tercile	6 (-6-18), 0.32	-0.5 (-4-3), 0.76	9 (-5-22), 0.20	0.2 (-0.1-0.6), 0.23	3 (-17), 0.14	**5 (2-8), 0.001**
Lymphocytes						
1st tercile	-	-	-	-	-	-
2nd tercile	**16 (5-28), 0.007**	3 (-0.2-6), 0.069	5 (-8-19), 0.44	**0.5 (-0.1-0.08), 0.008**	**4 (0.06-8), 0.047**	0.8 (-2-4), 0.62
3rd tercile	**18 (6-30), 0.004**	0.3 (-3-4), 0.86	9 (-5-23), 0.18	**0.4 (0.01-0.8), 0.042**	**5 (1-9), 0.010**	2 (-2-5), 0.34
Monocytes						
1st tercile	-	-	-	-	-	-
2nd tercile	-1 (-13-10), 081	-0.09 (-3-2), 0.96	0.3 (-13-14), 0.96	0.09 (-0.3-0.4), 0.61	4 (-0.4-7), 0.076	3 (-0.3-6),0.077
3rd tercile	8 (-4-19), 0.21	-2 (-5-2), 0.31	4 (-9-18), 0.52	0.3 (-0.07-0.7), 0.11	3 (-0.6-7), 0.093	**5 (2-8), 0.002**
Platelets						
1st tercile	-	-	-	-	-	-
2nd tercile	-4 (-16-7), 0.48	-2 (-5-1), 0.19	-4 (-18-9), 0.52	0.1 (-0.2-0.5), 0.50	0.4 (-3-4), 0.82	0.9 (-2-4), 0.54
3rd tercile	-9 (-21-3), 0.14	-2 (-6-0.9), 0.15	0.8 (-13-14), 0.91	**0.4 (0.07-0.8), 0.018**	4 (-0.04-8), 0.053	2 (-1-5), 0.18

	Alternative			Classical, alternative and lectin		
	%	Factor D		C3	C3a	Factor H x10e-3
Hemoglobin						
1st tercile	-	-		-	-	-
2nd tercile	5 (-6-17), 0.37	-626 (-1271-19), 0.057		13 (0.6-26), 0.041	3 (-0.02-6), 0.052	-126 (-366-115), 0.30
3rd tercile	8 (-4-21), 0.20	-403 (-1100-293), 0.25		12 (-1-26), 0.074	1 (-2-5), 0.44	-76 (-335-184), 0.57

Leucocytes					
1st tercile	-	-	-	-	-
2nd tercile	**8 (-4-19), 0.20**	**87 (-587-762), 0.80**	**17 (5-30), 0.007**	3 (0.06-6), 0.046	11 (-242-264), 0.93
3rd tercile	**18 (6-30), 0.004**	**678 (6-1350), 0.048**	**15 (2-28), 0.024**	2 (-1-6), 0.16	64 (-187-316), 0.62
Neutrophils					
1st tercile	-	-	-	-	-
2nd tercile	**3 (-9-15), 0.62**	182 (-496-859), 0.60	14 (1-26), 0.036	2 (-2-5), 0.34	-197 (-446-53), 0.12
3rd tercile	**13 (1-25), 0.030**	343 (-324-1011), 0.31	9 (-3-22), 0.14	3 (-0.3-6), 0.071	-41 (-286-205), 0.75
Lymphocytes					
1st tercile	-	-	-	-	-
2nd tercile	**18 (7-30), 0.002**	-405 (-1068-259), 0.23	**9 (-4-22), 0.17**	2 (-2-5), 0.31	-96 (-342-151), 0.44
3rd tercile	**24 (12-36), <0.001**	334 (-359-1027), 0.34	**17 (4-31), 0.010**	3 (-0.06-7), 0.055	141 (-116-399), 0.28
Monocytes					
1st tercile	-	-	-	-	-
2nd tercile	9 (-3-20),0.15	-77 (-723-569), 0.81	11 (-2-24), 0.086	0.2 (-3-3), 0.92	-71 (-313-170), 056
3rd tercile	10 (-2-22), 0.098	648 (-19-1314), 0.057	13 (-0.1-26), 0.052	3 (-0.7-6), 0.13	73 (-176-322), 0.57
Platelets					
1st tercile	-	-	-	-	-
2nd tercile	-4 (-15-8), 0.54	89 (-574-752), 0.79	9 (-3-21), 0.15	3 (-0.2-6), 0.071	54 (-192-299), 0.67
3rd tercile	-4 (-16-8), 0.48	162 (-513-838), 0.44	**13 (0.5-26), 0.041**	2 (-1-5), 0.27	10 (-240-259), 0.94

First tercile is considered the reference variable and complement functional test and proteins are the dependent variables. Significant p values are depicted in bold. Beta coefficients are adjusted for age, sex, SLICC and SLEDAI scores, and the intake of aspirin, prednisone, methotrexate, azathioprine and mofetil mycophenolate. SLEDAI covariable in this analysis does not contain the complement, thrombopenia, leukopenia and antiDNA items.

Supplementary Table S4. Multivariable relation of citopenias to the presence of anemia, leukopenia, neutropenia, lymphopenia and thrombocytopenia

	Anemia				Leukopenia				Neutropenia			
	No=231	Yes=39	p	p*	No= 218	Yes=52	p	p*	No= 255	Yes=15	p	p*
Classical pathway												
%	92 ± 2	84 ± 45	0.29	0.088	93 ± 38	83 ± 38	0.11	0.11	91 ± 38	87 ± 42	0.66	0.64
C1q, mg/dl	34 ± 10	32 ± 12	0.32	0.092	34 ± 10	33 ± 11	0.64	0.88	34 ± 11	33 ± 10	0.83	0.75
Lectin pathway												
%	33 (28-38)	32 (26-34)	0.76	0.58	13 (1-49)	6 (1-27)	0.12	0.17	12 (1-44)	10 (2-34)	0.49	0.44
Classical and alternative												
C2, mg/dl	**2.5 ± 1.1**	**2.2 ± 1.2**	**0.073**	**0.010**	2.5 ± 1.1	2.2 ± 1.1	0.12	0.18	2.4 ± 1.1	2.6 ± 1.5	0.62	0.77
C4, mg/dl	21 ± 12	19 ± 10	0.22	0.093	21 ± 12	19 ± 12	0.17	0.20	21 ± 12	24 ± 16	0.28	0.27
C1-inh, mg/dl	32 ± 9	30 ± 9	0.26	0.11	32 ± 9	30 ± 7	0.15	0.12	32 ± 9	28 ± 7	0.14	0.13
Alternative												
%	48 ± 38	45 ± 38	0.59	0.79	**50 ± 38**	**36 ± 35**	**0.014**	**0.022**	**49 ± 37**	**29 ± 39**	**0.050**	**0.046**
fD, ng/ml	2558 ± 1701	2544 ± 2468	0.97	0.94	2560 ± 1949	2539 ± 1092	0.95	0.53	2567 ± 1854	2335 ± 1029	0.70	0.57
Classical, alternative and lectin												
C3, mg/dl	132 ± 38	122 ± 49	0.17	0.051	**134 ± 41**	**115 ± 31**	**0.003**	**0.004**	131 ± 40	126 ± 38	0.63	0.69
C3a, mg/dl	39 ± 10	38 ± 12	0.74	0.23	**39 ± 10**	**35 ± 11**	**0..008**	**0.028**	39 ± 10	34 ± 10	0.097	0.051
fH, ng/ml	385 (291-559)	367 (227-579)	0.68	0.29	378 (280-550)	485 (302-640)	0.045	0.57	384 (284-559)	361 (269-623)	0.99	0.98

	Lymphopenia				Thrombocytopenia			
	No=229	Yes=41	p	p*	No=238	Yes=32	p	p*
Classical pathway								
%	**93 ± 37**	**82 ± 42**	**0.11**	**0.033**	90 ± 39	96 ± 33	0.45	0.67
C1q, mg/dl	34 ± 10	35 ± 13	0.53	0.64	34 ± 11	35 ± 10	0.63	0.41
Lectin pathway								
%	9 (1-45)	21 (1-38)	0.51	0.99	9 (1-40)	27 (1-57)	0.18	0.27
Classical and alternative								
C2, mg/dl	**2.5 ± 1.2**	**2.2 ± 1.1**	**0.081**	**0.037**	2.5 ± 1.2	2.1 ± 1.1	0.057	0.050
C4, mg/dl	22 ± 12	17 ± 13	0.045	0.14	**21 ± 12**	**18 ± 13**	**0.21**	**0.049**
C1-inh, mg/dl	32 ± 9	33 ± 6	0.65	0.98	32 ± 9	32 ± 9	0.83	0.59

Alternative								
%	51 ± 37	28 ± 33	<0.001	0.003	47 ± 38	52 ± 36	0.45	0.67
fD, ng/ml	2509 ± 1713	2863 ± 2437	0.34	0.60	2549 ± 1905	2604 ± 1131	0.88	0.92
Classical, alternative and lectin								
C3, mg/dl	**133 ± 41**	**114 ± 33**	**0.007**	**0.009**	**132 ± 41**	**116 ± 34**	**0.038**	**0.004**
C3a, mg/dl	39 ± 10	37 ± 12	0.40	0.26	39 ± 10	37 ± 8	0.49	0.45
fH, ng/ml	381 (276-559)	465 (300-595)	0.79	0.97	382 (287-564)	474 (234-559)	0.70	0.69

* Adjusted for age, sex, SLICC-DI (damage) and SLEDAI (activity) scores, as well as the use of aspirin, prednisone, methotrexate, azathioprine, and mycophenolate mofetil. Anemia is defined as hemoglobin <11.9 g/dL (119 g/L) or hematocrit <35 percent in females and hemoglobin <13.6 g/dL (136 g/L) or hematocrit <40 percent in males. Leukopenia: leucocytes <4000 cells/microl, neutropenia: neutrophils <1500 cells/microl, lymphopenia: lymphocytes <1000 cells/microl, thrombocytopenia: platelets <1500 cells/microl. Significant p values are depicted in bold.
